# Elevated Risk of Herpes Zoster in Vitiligo Patients: A Nationwide Population‐Based Cohort Study of Taiwan

**DOI:** 10.1111/1346-8138.70140

**Published:** 2026-01-09

**Authors:** Bing‐Sian Lin, Chi‐Hsiang Chung, Tsu‐Hsuan Weng, Chun‐Teng Tsai, Sheng‐Wen Liu, Wu‐Chien Chien, Chih‐Tsung Hung

**Affiliations:** ^1^ Department of Dermatology Tri‐Service General Hospital, National Defense Medical University Taipei City Taiwan; ^2^ School of Medicine National Defense Medical University Taipei City Taiwan; ^3^ Department of Medical Research Tri‐Service General Hospital, National Defense Medical University Taipei City Taiwan; ^4^ School of Public Health National Defense Medical University Taipei City Taiwan; ^5^ Taiwanese Injury Prevention and Safety Promotion Association (TIPSPA) Taipei City Taiwan

**Keywords:** herpes zoster, herpes zoster vaccine, Janus kinase inhibitors, steroids, vitiligo

## Abstract

Emerging therapies for vitiligo, such as Janus kinase (JAK) inhibitors, have raised concerns about an increased risk of herpes zoster (HZ), emphasizing the need to clarify the baseline HZ risk in patients with vitiligo. This study aimed to determine whether vitiligo itself is associated with a higher risk of HZ and to identify potential modifying factors, including systemic treatments. Using Taiwan's Longitudinal Health Insurance Database from 2010 to 2022, a retrospective nationwide cohort study was conducted. Patients with vitiligo were identified through diagnostic codes and matched in a 1:4 ratio with non‐vitiligo controls by age, gender, index date, and comorbidities using propensity score matching. Subgroup analyses evaluated HZ risk among patients receiving systemic treatments, including phototherapy and immunosuppressants. A total of 79 910 individuals were included after matching. During the study period, the incidence of herpes zoster was significantly higher in patients with vitiligo than in controls (7.78% vs. 2.72%, *p* < 0.001). After adjusting for potential confounders, vitiligo remained independently associated with an increased risk of HZ (adjusted hazard ratio [aHR]: 1.532). The risk of HZ increased with age and was higher among female patients. Subgroup analysis further revealed that vitiligo patients receiving systemic therapy had the greatest susceptibility to HZ, especially those treated with cyclosporine (aHR: 1.891), methotrexate (aHR: 1.981), and systemic corticosteroids (aHR: 1.474). In conclusion, this large population‐based study demonstrates that vitiligo is an independent risk factor for herpes zoster, and systemic immunosuppressive therapies further augment this risk. Clinicians should be aware of the potentially increased vulnerability to HZ among patients with vitiligo, particularly in older or female individuals. These findings may help inform general clinical considerations regarding preventive strategies, including herpes zoster vaccination, to reduce the risk of infection‐related complications in this population.

## 1 Introduction

Vitiligo is a common autoimmune condition characterized by abnormal T‐cell‐mediated attacks on melanocytes, resulting in asymptomatic depigmented patches. Its prevalence is estimated at 0.5%–1% [1]. Although vitiligo typically does not cause physical discomfort, it can lead to significant psychological distress, social stigma, and even discrimination [2]. Therefore, providing effective treatment is a key responsibility for dermatologists.

Herpes zoster (HZ) is another prevalent skin condition, with a lifetime risk of 10%–30% [3]. It manifests as painful grouped vesicles and may result in chronic neuropathic pain, particularly affecting adults and the elderly. This leads to a considerable societal burden, including reduced workforce productivity and increased healthcare utilization [4].

Recent advances in dermatological therapeutics have introduced novel treatment options, particularly Janus kinase inhibitors (JAKi) [5], which have shown efficacy in treating atopic dermatitis [6] and alopecia areata [7]. However, these treatments are associated with an increased risk of HZ infection. While JAKi therapies for vitiligo are currently under clinical investigation, it is crucial to establish baseline HZ risk in vitiligo patients before these treatments become widely available. Understanding this baseline risk will be essential for developing appropriate risk mitigation strategies, including prophylactic vaccination protocols, to minimize potential adverse effects when these novel therapies are implemented in vitiligo management.

To date, the risk of HZ in patients with vitiligo has not been thoroughly examined using real‐world data. In this study, we conducted a longitudinal cohort analysis using the National Health Insurance Research Database (NHIRD) to explore the association between vitiligo and HZ.

## 2 Methods

### 2.1 Source of Data

Data from the Longitudinal Health Insurance Database (LHID), covering 2010–2022, were utilized for the retrospective, nationwide cohort study. The LHID, a subset of the NHIRD, is maintained by Taiwan's National Health Insurance (NHI) program. Established in 1995, the program provides healthcare coverage to over 99% of Taiwan's population, which exceeds 23 million individuals. Personal information in the database is encrypted to ensure privacy and ethical compliance.

The LHID provides detailed information, including diagnostic and procedural codes based on the International Classification of Diseases, Ninth Revision, Clinical Modification (ICD‐9‐CM) (until 2014) and ICD‐10‐CM (after 2015). The LHID also provides demographic data such as gender and birthdate, as well as admission and discharge dates, and clinical outcomes. The database includes a randomly chosen sample of about one million people, accounting for about 5% of the total population of Taiwan.

To ensure the reliability of the data, the National Health Insurance Administration collaborates with external experts to evaluate outpatient and inpatient claim records. Consequently, the NHIRD serves as a reliable database for exploring the association between vitiligo and the risk of HZ.

### 2.2 Patient Selection and Study Design

We analyzed vitiligo patients from 2010 to 2022 (the index period), with the index date defined as the first recorded healthcare claim for vitiligo during this period. The inclusion criteria for vitiligo patients were defined as having either three or more outpatient department (OPD) diagnoses or at least one hospitalization with the ICD‐9‐CM code 709.01 or ICD‐10‐CM code L80. Exclusion criteria included a prior diagnosis of vitiligo before 2010, having HZ before tracking, age below 20 years, and patients with diagnostic codes for diseases mimicking vitiligo, including pityriasis versicolor (ICD‐9‐CM code 111.0 or ICD‐10‐CM code B36), pityriasis alba (ICD‐9‐CM code 696.5 or ICD‐10‐CM code L30.5), and morphea (ICD‐9‐CM code 701.0 or ICD‐10‐CM code L94).

After participants matched with age and gender, a retrospective cohort study incorporating propensity score matching was conducted. The main purpose is to assess whether vitiligo patients have increased risk for HZ. Eligible patients with vitiligo were identified from the outlined data sources and grouped into the vitiligo cohort. This cohort was matched in a 1:4 ratio with a control group randomly drawn from the remaining LHID population. The control group was paired with the study group by age, gender, index date, and Charlson Comorbidity Index (CCI). Since autoimmune diseases were already incorporated as covariates in the CCI, further adjustment for autoimmune disease was not performed to avoid overadjustment. Exclusion criteria were the same as the vitiligo cohort.

**FIGURE 1:**
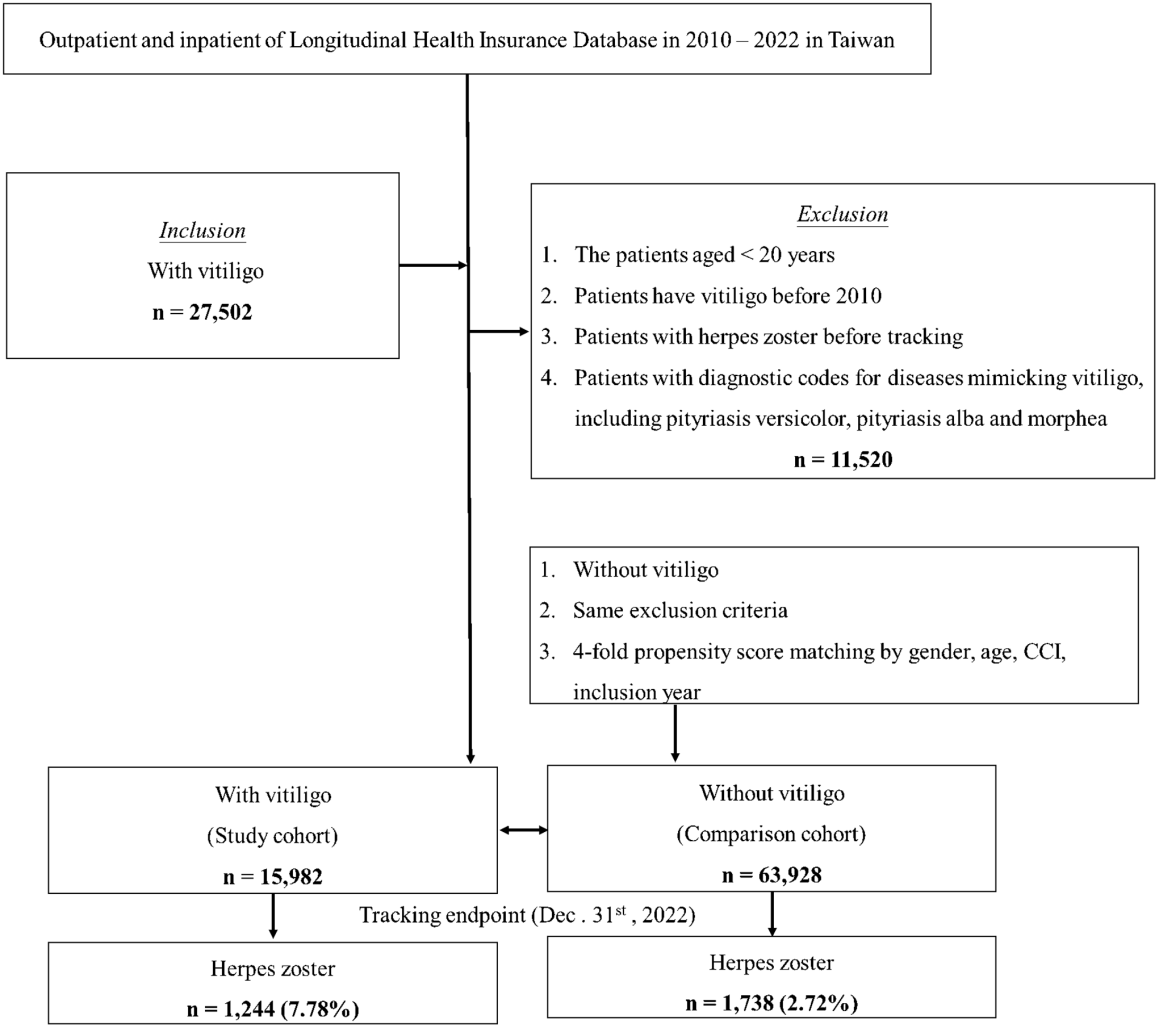
Flow chart of recruitment of study cohort.

Additionally, the vitiligo cohort was categorized based on the different systemic treatments they received, and further subgroup analyses were performed. The systemic treatment was defined as receiving one of the following treatments including phototherapy, systemic steroid, Methotrexate, Azathioprine, and Cyclosporine. Patients receiving phototherapy were identified with National Health Insurance codes: 51018B, 51019B. Those receiving corticosteroids were identified with ATC codes: H02AB01, H02AB02, H02AB04, H02AB06, H02AB09, H02AB10. Those receiving methotrexate were identified with ATC codes: L04AX03. Those receiving azathioprine were identified with ATC codes: L04AX01. Those receiving cyclosporine were identified with ATC codes: L04AD01. Figure 1 outlines the selection process for the study sample.

### 2.3 Outcome Measurements

Patients are tracked from the date of inclusion until the onset of one of the following: HZ diagnosis, death, withdrawal from the National Health Insurance (NHI) program, or the study's conclusion in late 2022. HZ was identified when a patient had either two or more OPD diagnoses or one hospitalization for the condition within a year, classified under ICD‐9‐CM code 053 or ICD‐10‐CM code B02.

### 2.4 Statistical Analysis

This study employed a propensity score‐matching approach, utilizing a Cox regression model to account for potential confounders. These factors included index year, gender, age, and CCI. A tolerance threshold of 0.15 was applied, and the nearest neighbor method was used with a 1:4 matching ratio between the study and control groups. Continuous variables were analyzed by calculating their means and standard deviations (SD), with comparisons made using *t*‐tests. The chi‐squared test or Fisher's exact test was employed to calculate percentages for categorical data.

The relationship between clinical features and outcomes of interest was analyzed using the Cox regression model. The cumulative risk of HZ in the subgroup analysis of different vitiligo patients compared to the control group was assessed via the log‐rank test and represented using Kaplan–Meier curves. Hazard ratios (HRs) and 95% confidence intervals (CIs) were reported, with a significance level set at *p <* 0.05. All analyses were conducted using IBM SPSS version 22.0.

### 2.5 Ethic

Ethical clearance for this study was granted by the Institutional Review Board of Tri‐Service General Hospital, National Defense Medical University, Taipei, Taiwan (approval number TSGHIRB E202516047, dated August 3, 2025). As the Taiwan NHIRD contains anonymized secondary data, the board waived the requirement for individual informed consent. All study procedures conformed to the principles set forth in the Declaration of Helsinki and complied with applicable ethical and regulatory standards.

## 3 Results

### 3.1 Characteristics of the Study

As shown in Table 1, this cohort study included 79 910 participants, comprising 15 982 individuals with vitiligo (20.00%) and 63 928 without vitiligo (80.00%). The control group was matched to the vitiligo group by age, gender, index date, and Charlson Comorbidity Index (CCI), ensuring comparability at baseline.

**TABLE 1:** Characteristics of study.

Vitiligo	Total		With		Without		*p*
Variables	*n*	%	*n*	%	*n*	%	
Total	79 910		15 982	20.00	63 928	80.00	
Herpes zoster							< 0.001
Without	76 928	96.27	14 738	92.22	62 190	97.28	
With	2982	3.73	1244	7.78	1738	2.72	
Gender							1.000
Male	33 730	42.21	6746	42.21	26 984	42.21	
Female	46 180	57.79	9236	57.79	36 944	57.79	
Age (years)	50.69 ± 15.74		50.69 ± 15.74		50.69 ± 15.74		1.000
Age groups (years)							1.000
20–30	9655	12.08	1931	12.08	7724	12.08	
31–40	13 325	16.68	2665	16.68	10 660	16.68	
41–50	15 595	19.52	3119	19.52	12 476	19.52	
51–60	17 965	22.48	3593	22.48	14 372	22.48	
≧ 60	23 370	29.25	4674	29.25	18 696	29.25	
CCI	0.89 ± 1.75		0.89 ± 1.75		0.89 ± 1.75		1.000

*Note:* *p*, Chi‐square/Fisher exact test on category variables and *t*‐test on continue variables. The shading indicates statistically significant results with *p* < 0.05.

Following matching, gender distribution was identical across both groups: 42.21% male (6746 with vitiligo; 26 984 without) and 57.79% female (9236 with vitiligo; 36 944 without), with a *p*‐value of 1.000 confirming no significant difference. The mean age was 50.69 ± 15.74 years in both groups. Age distribution was as follows: 20–30 years (12.08%), 31–40 years (16.68%), 41–50 years (19.52%), 51–60 years (22.48%), and ≥ 60 years (29.25%), with no notable differences between cohorts. The mean CCI score was also identical (0.89 ± 1.75), again with no significant difference (*p* = 1.000), confirming effective matching for comorbidity burden.

Table 1 also summarizes the occurrence of HZ in the endpoint of the cohort study. Overall, 2982 participants (3.73%) developed HZ during follow‐up. The incidence was significantly higher in the vitiligo group (7.78%, *n* = 1244) compared to the control group (2.72%, *n* = 1738, *p* < 0.001) (Table S1).

### 3.2 HRs of HZ With Vitiligo With Cox Regression

Table 2 presents the results of the Cox regression analysis evaluating both crude and adjusted hazard ratios (HRs) for HZ in individuals with and without vitiligo, controlling for relevant covariates. In the unadjusted model, vitiligo was associated with a significantly increased risk of HZ (crude HR (cHR): 1.605; 95% CI: 1.488–1.731; *p <* 0.001). Female gender also showed a higher risk compared to males (cHR: 1.212; 95% CI: 1.125–1.305; *p <* 0.001). The risk increased progressively with age, using the 20–30‐year group as reference: [8, 9, 10, 11, 12, 13, 14, 15, 16, 17] years: cHR 1.212 (95% CI: 0.994–1.477; *p =* 0.058). 41–50 years: cHR 2.260 (95% CI: 1.889–2.703; *p =* 0.019). 51–60 years: cHR 3.582 (95% CI: 3.025–4.241; *p <* 0.001). ≥ 60 years: cHR 4.505 (95% CI: 3.823–5.309; *p <* 0.001).

**TABLE 2:** Factors of herpes zoster by using Cox regression.

Variables	Crude HR	95% CI	95% CI	*p*	Adjust HR	95% CI	95% CI	*p*
Vitiligo								
Without	Reference				Reference			
With	1.605	1.488	1.731	< 0.001	1.532	1.419	1.655	< 0.001
Gender								
Male	Reference				Reference			
Female	1.212	1.125	1.305	< 0.001	1.219	1.131	1.314	< 0.001
Age groups (years)								
20–30	Reference				Reference			
31–40	1.212	0.994	1.477	0.058	1.154	0.946	1.408	0.157
41–50	2.260	1.889	2.703	0.019	2.063	1.724	2.469	< 0.001
51–60	3.582	3.025	4.241	< 0.001	3.173	2.676	3.762	< 0.001
≧ 60	4.505	3.823	5.309	< 0.001	3.812	3.225	4.507	< 0.001

*Note:* The shading indicates statistically significant results with *p* < 0.05.

Abbreviations: aHR, Adjusted hazard ratio: Adjusted variables listed in the table; CI, confidence interval.

After adjusting for gender and age, vitiligo remained an independent risk factor (adjusted HR (aHR): 1.532; 95% CI: 1.419–1.655; *p <* 0.001), indicating a 53.2% higher risk of HZ compared to individuals without vitiligo. The adjusted analysis also confirmed female gender as a significant risk factor (aHR: 1.219; 95% CI: 1.131–1.314; *p <* 0.001), along with a continued age‐dependent increase in risk: 31–40 years: aHR 1.154 (95% CI: 0.946–1.408; *p =* 0.157). 41–50 years: aHR 2.063 (95% CI: 1.724–2.469; *p <* 0.001). 51–60 years: aHR 3.173 (95% CI: 2.676–3.762; *p <* 0.001). ≥ 60 years: aHR 3.812 (95% CI: 3.225–4.507; *p <* 0.001).

### 3.3 Kaplan–Meier Analysis for Accumulative Risk of HZ

**FIGURE 2:**
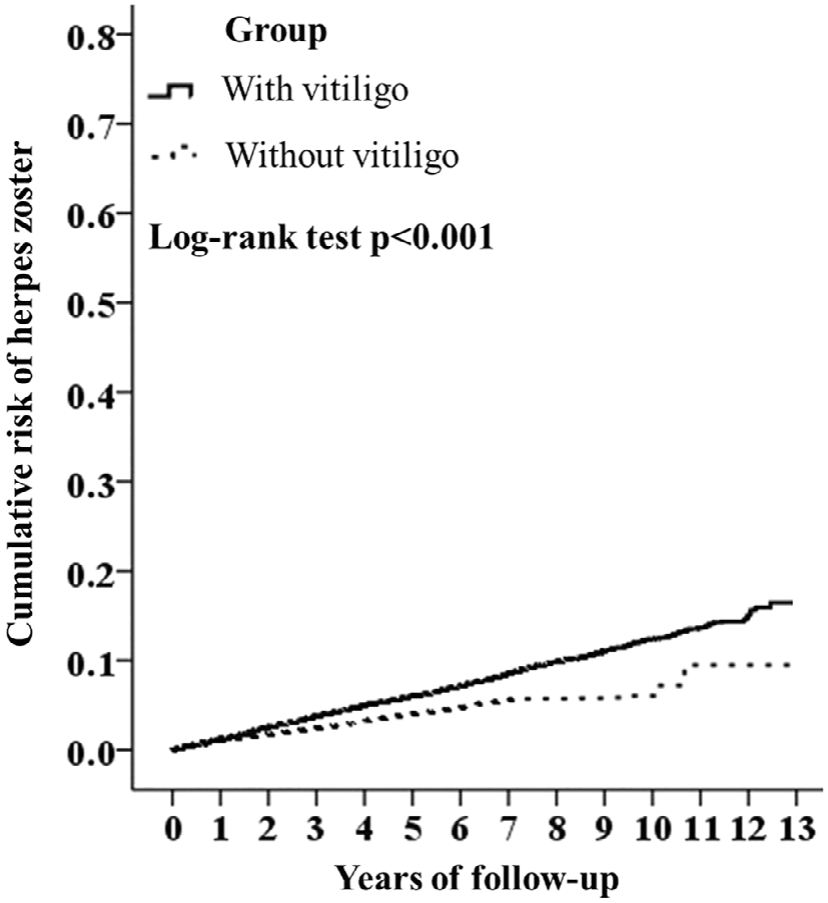
Kaplan meier plot for cumulative risk of herpes zoster in patients with and without vitiligo.

The Figure 2 shows the result of Kaplan–Meier Analysis for accumulative risk of HZ in patients with and without vitiligo. It shows patients with vitiligo have statistically significant higher risk of HZ, and this trend persists in the entire study course (*p <* 0.001).

### 3.4 Subgroup Analysis of Different Therapy and Risk of HZ

Table 3 summarizes the crude and adjusted hazard ratios for HZ among vitiligo patients by treatment modality. Compared with the non‐vitiligo group (*n* = 63 928; incidence rate: 430.85 per 100 000 PYs), vitiligo patients receiving systemic therapy (*n* = 2643) had 271 events (incidence: 1737.88 per 100 000 PYs), showing the highest risk (aHR: 2.117; 95% CI: 1.846–2.428; *p* < 0.001). Those without systemic therapy (*n* = 13 339) experienced 973 events (incidence: 1236.34 per 100 000 PYs) and also had a significantly increased risk (aHR: 1.419; 95% CI: 1.312–1.535; *p* < 0.001). Overall, both subgroups demonstrated elevated HZ risk, with systemic‐therapy users most affected.

**TABLE 3:** Hazard ratio of Herpes zoster infection in vitiligo subgroup receiving different treatment.

Cohort	Population	Events	PYs	Rate	Crude HR	95% CI	95% CI	*p*	Adjusted HR	95% CI	95% CI	*p*
With and without systemic therapy and non‐vitiligo												
Vitiligo‐with systemic therapy	2643	271	15593.70	1737.88	2.096	1.827	2.404	< 0.001	2.117	1.846	2.428	< 0.001
Vitiligo‐without systemic therapy	13 339	973	78700.10	1236.34	1.507	1.393	1.630	< 0.001	1.419	1.312	1.535	< 0.001
Without vitiligo	63 928	1738	403385.68	430.85	Reference				Reference			
Vitiligo with systemic therapy												
Phototherapy												
Without	15 779	1221	96251.90	1268.55	Reference				Reference			
With	203	23	1197.70	1920.35	1.081	0.715	1.633	0.713	1.168	0.773	1.766	0.460
Systemic steroids												
Without	13 735	1014	90651.00	1118.58	Reference				Reference			
With	2247	230	12133.80	1895.53	1.361	1.179	1.570	< 0.001	1.474	1.277	1.702	< 0.001
Methotrexate												
Without	15 731	1215	92812.90	1309.09	Reference				Reference			
With	251	29	1531.10	1894.06	1.686	1.167	2.437	0.005	1.981	1.370	2.865	< 0.001
Azathioprine												
Without	15 783	1226	99432.90	1232.99	Reference				Reference			
With	199	18	1134.30	1586.88	1.227	0.770	1.953	0.390	1.475	0.926	2.351	0.102
Cyclosporine												
Without	15 883	1232	90533.10	1360.83	Reference				Reference			
With	99	12	524.70	2287.02	1.766	1.000	3.118	0.050	1.891	1.070	3.340	0.028

Abbreviations: Adjusted HR, Adjusted Hazard ratio: Adjusted for the variables listed in Table 3; CI, confidence interval; PYs, Person‐years.

Treatment‐specific analyses showed no significant association with phototherapy (*n* = 203; incidence: 1920.35 per 100 000 PYs; aHR: 1.168; *p* = 0.460). Systemic steroid users (*n* = 2247; incidence: 1895.53 per 100 000 PYs) had a significantly higher risk (aHR: 1.474; *p* < 0.001). Methotrexate users (*n* = 251; incidence: 1894.06 per 100 000 PYs) also showed increased risk (aHR: 1.981; *p* < 0.001). Azathioprine users (*n* = 199; incidence: 1586.88 per 100 000 PYs) did not show a statistically significant association (aHR: 1.475; *p* = 0.102). Cyclosporine users (*n* = 99; incidence: 2287.02 per 100 000 PYs) exhibited the highest treatment‐specific risk (aHR: 1.891; *p* = 0.028).

## 4 Discussion

Our study demonstrated that vitiligo patients had a significantly higher risk of HZ compared to those without vitiligo (adjusted HR = 1.532; 95% CI: 1.419–1.655; *p <* 0.001). Female gender and advancing age were also identified as independent risk factors, consistent with previous studies [18, 19]. Subgroup analysis further revealed that even vitiligo patients not receiving systemic treatment exhibited an elevated risk of HZ compared to non‐vitiligo controls, suggesting that the diagnosis of vitiligo itself may be a risk factor.

One previous study [20] provided a comprehensive comparison of comorbidities across various skin diseases. In that analysis, the vitiligo cohort demonstrated a higher incidence rate of HZ; however, the confidence intervals overlapped between the vitiligo and non‐vitiligo groups (851.5 [95% CI: 712.0–1010.5] vs. 656.3 [95% CI: 534.6–797.5]), suggesting that the difference was not statistically significant. We also note that the earlier study included 8912 patients with vitiligo, whereas our study enrolled 15 982. It is possible that the smaller sample size in the previous work may have limited its statistical power to detect a significant association. Furthermore, a cohort study conducted in patients with end‐stage renal disease (ESRD) reported an increased risk of HZ among individuals with vitiligo (adjusted relative risk, 1.51; *p* = 0.004), although we acknowledge that findings from ESRD populations may not be fully generalizable to the broader population [21].

The development of HZ is influenced by the interaction between host immunity and latent varicella‐zoster virus. In particular, cell‐mediated immunity plays a critical role in controlling viral reactivation [22], and immunosuppression is a well‐established contributor to increased HZ risk [23]. Besides, it has been well established that vitiligo can cause substantial mental and social stress and reduce quality of life to patients [2]. Psychological stress has been studied to have association with increased risk of HZ [24]. Thus, although the exact mechanism linking vitiligo to HZ remains unclear, we hypothesize that psychological stress may contribute to impaired cellular immunity [25], thereby increasing susceptibility to HZ. Previous studies have reported a reduced risk of melanoma and nonmelanoma skin cancer in patients with vitiligo [26], which may be attributed to enhanced immune surveillance against cutaneous malignancies in this population [27]. In contrast, our study observed an increased risk of HZ, suggesting that antitumor and antiviral immune responses in vitiligo may not be uniformly affected. Reactivation of varicella‐zoster virus (VZV) has been associated with decreased levels of circulating VZV‐specific memory T cells, leading to impaired viral control within the dorsal root ganglia and subsequent viral spread to the skin [28]. In patients with vitiligo, alterations in antiviral immune function may be influenced by immune exhaustion, as persistent immune activation has been shown to contribute to functional impairment of specific T‐cell subsets [29]. Overall, while cutaneous immune surveillance in vitiligo may be relatively enhanced, this localized immune activity may not necessarily correspond to a parallel enhancement of systemic antiviral immunity against VZV. These interpretations remain speculative and underscore the need for further mechanistic and longitudinal studies.

As for patients receiving systemic treatment experience higher risk of HZ, we speculated that treatment‐related immunosuppression may be a contributing factor. Vitiligo management includes phototherapy (Narrowband UVB (NB‐UVB), photochemotherapy, excimer), topical agents (steroids, calcineurin inhibitors, JAKi), and systemic therapies (systemic steroids, methotrexate, azathioprine, cyclosporine, and JAKi) [30, 31]. Many of these treatments, particularly systemic therapies, can suppress immune function. Therefore, we conducted a subgroup analysis to evaluate the impact of various systemic treatments on HZ risk in patients with vitiligo.

Our study found no increased risk of HZ in vitiligo patients receiving phototherapy. NB‐UVB phototherapy has been shown to suppress cell‐mediated immune responses by inhibiting effector T‐cell activity in the skin [32, 33]. Interestingly, a previous study in psoriasis patients reported a reduced risk of HZ following UV phototherapy, possibly due to UV‐induced vitamin D synthesis, which may enhance T cell–mediated immunity [34, 35]. It is possible that in vitiligo patients, the immunosuppressive and immunostimulatory effects of NB‐UVB counterbalance each other, resulting in no significant impact on HZ risk. However, further research is needed to clarify the underlying mechanisms. In contrast, vitiligo patients treated with systemic steroids, methotrexate, or cyclosporine exhibited a significantly increased risk of developing HZ. Glucocorticoids impair cell‐mediated immunity by downregulating adhesion molecule expression, reducing T‐cell migration, and inducing peripheral T‐cell apoptosis [36]. These effects contribute to immune suppression and increased susceptibility to HZ. One population‐based study even demonstrated that a single dose of systemic corticosteroids can elevate HZ risk [37]. Methotrexate (MTX), a folate antagonist and disease‐modifying antirheumatic drug (DMARD), is commonly used to treat autoimmune and inflammatory diseases such as psoriasis, rheumatoid arthritis, and inflammatory bowel disease. It suppresses T‐cell proliferation and reduces TNF‐α activity, which is elevated in vitiligo lesions [38, 39]. Consequently, MTX has gained attention as a potential treatment for vitiligo. However, data regarding HZ risk with MTX use are inconsistent [8, 40]. While some population‐based studies in psoriasis patients reported no increased risk—or even a reduced risk—of HZ with MTX monotherapy [34], our findings contradict this, showing an elevated HZ risk among vitiligo patients receiving MTX. This discrepancy suggests that factors beyond MTX's immunosuppressive properties may contribute to the increased risk, warranting further investigation. Cyclosporine, a calcineurin inhibitor, suppresses T‐cell activation and inhibits CD8+ T cells, which are implicated in vitiligo pathogenesis [9]. It has shown efficacy in stabilizing active vitiligo [10]. However, its immunosuppressive action may also predispose patients to HZ, as observed in our analysis. Azathioprine, a purine analog that interferes with DNA and RNA synthesis, also acts as an immunosuppressant. It has been reported to slow vitiligo progression and enhance the re‐pigmentary effects of UV therapy [11, 12]. While our study found a trend toward increased HZ risk in patients using azathioprine, the association did not reach statistical significance. Prior studies in inflammatory bowel disease patients have identified azathioprine as a risk factor for HZ after multivariable adjustment [13, 14]. Nevertheless, in the subgroup analysis, those treated with phototherapy, azathioprine, and cyclosporine accounted for only 1.3%, 1.26%, and 0.62% of the cohort, respectively. These groups showed wide confidence intervals around the hazard ratio, and the statistical power of these subgroup analyses was limited. Thus, the results should be interpreted with caution. Larger studies are needed to clarify this potential association in the vitiligo population. In addition, to clarify the potential impact of multiple treatments, a dedicated subgroup analysis regarding systemic combination therapy was performed, analyzing these patients as an independent subgroup. Our data (Table S2) indicated a non‐significant trend toward an elevated risk of HZ in vitiligo patients receiving two or three systemic therapies when compared to those receiving only one. While not statistically conclusive, this observation suggests that the effect of cumulative systemic exposure on HZ risk may warrant further exploration.

Understanding the risk of herpes zoster (HZ) in vitiligo patients has become increasingly important with the advent of JAKis as emerging treatment options. Several JAKis have shown promising results: Upadacitinib demonstrated clinical efficacy in patients with extensive non‐segmental vitiligo in a phase 2 trial [15]; Baricitinib, when combined with NB‐UVB, significantly outperformed NB‐UVB monotherapy in patients with active vitiligo [16]; Povorcitinib yielded substantial repigmentation in a phase 2 study [17]; and Deucravacitinib has shown potential in halting depigmentation and is currently under clinical investigation (ClinicalTrials.gov Identifier: NCT06327321). Our study provides real‐world evidence of an elevated baseline risk of HZ among vitiligo patients. Given that multiple studies have reported increased HZ risk associated with JAKi use [41], understanding the baseline risk in patients with vitiligo is helpful for putting these safety concerns into context. Previous research has shown that zoster vaccines—both the recombinant zoster vaccine (RZV) and the live zoster vaccine (ZVL)—can lower the chance of developing HZ and its complications [42]. Although our observational study cannot establish causality, these results may support general clinical discussions about HZ prevention, especially when systemic treatments are being considered for vitiligo. Besides, herpes zoster vaccination could potentially affect our findings. Nevertheless, the HZ vaccine was only introduced in Taiwan in 2013 and is not covered by the NHI, resulting in relatively low vaccination rate. Therefore, the potential bias introduced by HZ vaccination is expected to be minimal in this study.

However, our study has several limitations. First, the severity and subtype of vitiligo could not be evaluated in this study because the NHIRD does not include these clinical details. Variations in disease severity may affect underlying immune status and could therefore act as unmeasured confounders in the observed association between vitiligo and HZ risk. Second, the use of the NHIRD limited access to detailed clinical data, such as vitiligo subtype, disease severity, and patient lifestyle factors (e.g., alcohol or tobacco use), all of which may influence treatment decisions and HZ risk. Third, our study population consisted predominantly of Taiwanese patients, which may limit the generalizability of our findings to other ethnic populations. Given that the risk of herpes zoster, vitiligo prevalence, clinical presentation, and treatment responses can vary significantly across different racial and ethnic groups, caution should be exercised when extrapolating these results to non‐Asian populations [43, 44, 45, 46].

## 5 Conclusion

Our findings demonstrate a significantly increased risk of HZ in patients with vitiligo (aHR = 1.532; 95% CI: 1.419–1.655; *p <* 0.001), suggesting that vitiligo itself is an independent risk factor, regardless of treatment status. Clinicians should be aware of this elevated risk, and vaccination against HZ may be considered for patients with vitiligo to reduce the incidence and potential complications of the disease.

## Conflicts of Interest

The authors declare no conflicts of interest.

## Supporting information


**Table S1:** Events of herpes zoster in the endpoint.
**Table S2:** Hazard ratio of Herpes zoster infection in vitiligo subgroup receiving different number of treatments.
**Table S3:** presents additional analyses derived from the same study cohort and dataset described in the Methods section.

## Data Availability

The data that support the findings of this study are available on request from the corresponding author. The data are not publicly available due to privacy or ethical restrictions.
